# Effect of a novel two-desk sit-to-stand workplace (ACTIVE OFFICE) on sitting time, performance and physiological parameters: protocol for a randomized control trial

**DOI:** 10.1186/s12889-016-3271-y

**Published:** 2016-07-15

**Authors:** Bernhard Schwartz, Jay M. Kapellusch, Andreas Schrempf, Kathrin Probst, Michael Haller, Arnold Baca

**Affiliations:** Department of Medical Engineering, University of Applied Sciences Upper Austria, Garnisonstrasse 21, 4020 Linz, Austria; Department of Sport Science, University of Vienna, Auf der Schmelz 6, 1150 Vienna, Austria; Department of Occupational Science & Technology, University of Wisconsin – Milwaukee, P.O. Box 413, Milwaukee, WI 53201 USA; Media Interactive Lab, University of Applied Sciences Upper Austria, Softwarepark 11, 4232 Hagenberg, Austria

**Keywords:** Postural changes, Standing, Sitting, Cognitive performance, Reaction time, Concentration, Workload, Office, Stroop-test, d2R-test of attention

## Abstract

**Background:**

Prolonged sitting is ubiquitous in modern society and linked to several diseases. Height-adjustable desks are being used to decrease worksite based sitting time (ST). Single-desk sit-to-stand workplaces exhibit small ST reduction potential and short-term loss in performance. The aim of this paper is to report the study design and methodology of an ACTIVE OFFICE trial.

**Design:**

The study was a 1-year three-arm, randomized controlled trial in 18 healthy Austrian office workers. Allocation was done via a regional health insurance, with data collection during Jan 2014 – March 2015. Participants were allocated to either an intervention or control group. Intervention group subjects were provided with traditional or two-desk sit-to-stand workstations in either the first or the second half of the study, while control subjects did not experience any changes during the whole study duration.

Sitting time and physical activity (IPAQ-long), cognitive performance (text editing task, Stroop-test, d2R test of attention), workload perception (NASA-TLX) and physiological parameters (salivary cortisol, heartrate variability and body weight) were measured pre- and post-intervention (23 weeks after baseline) for intervention and control periods. Postural changes and sitting/standing time (software logger) were recorded at the workplace for the whole intervention period.

**Discussion:**

This study evaluates the effects of a novel two-desk sit-to-stand workplace on sitting time, physical parameters and work performance of healthy office based workers. If the intervention proves effective, it has a great potential to be implemented in regular workplaces to reduce diseases related to prolonged sitting.

**Trial registration:**

ClinicalTrials.gov Identifier: NCT02825303, July 2016 (retrospectively registered).

## Background

Prolonged sitting is ubiquitous in modern society and the amount of physically inactive people is rising in many countries [1, 2]. Ongoing computerization is a main cause for changes in physical activity and sitting time patterns [3, 4]. Screen time, which is commonly associated with sitting, has been dramatically increased by a rising prevalence of computers in school and occupational environments [4]. Duration of sitting time has also been shown to increase with age [5].

In 2013, 11 % of all European citizens (aged 14 years and older) spent more than 8.5 h per day in a sitting posture [6]. In the working age population, white-collar workers are most frequently affected by this amount of sitting time (21 %) and exhibit a more than four times higher risk of being exposed to prolonged sitting in comparison to manual occupations [6]. Especially office workers and call center employees are affected by prolonged sitting periods. The total amount of sitting in these occupations can exceed more than 80 % of the working day [7, 8].

Prolonged sitting is a risk factor for cardiovascular and musculoskeletal diseases, diabetes, several types of cancer and all-cause mortality [9–14]. In combination with static and awkward postures, the prevalence of musculoskeletal diseases (e.g. back pain, chest pain) can increase further [14]. As additional physical activity cannot fully compensate the effects of prolonged sitting [15, 16], standing between prolonged sitting periods and reduction of sedentary pursuits should be a goal for adults, irrespective of their exercise habits [17].

Given that most of the world’s population spend averagely one third of their adult life at work [18], it seems clear that worksite based interventions for reducing sitting time are key elements of daily sitting time reduction. Generally, worksite based recommendations in offices contain “*Sit less”, “Stand up”, “Move more” and “Change postures regularly*” [16, 19, 20]. In order to fulfill postural recommendations different types of worksite based interventions have been started. Besides numerous activity promotion programs, which typically replace sitting time with low-intensity physical activity [21], the implementation of sit-to-stand or active workstations is commonly used to diminish occupational sitting time [22].

Large differences in sitting time reduction have been found for different types of sit-to-stand workstations [22]. While non-significant changes in sitting time for sit-to-stand desk users in open plan offices occurred [23], meta-analysis showed an average reduction in sitting time of 77 min per 8-h workday for activity-permissive workstations [22]. Multi-component interventions (e.g. management consultation) can further enhance this effect [24].

Although the implementation of sit-to-stand or active workstations can help to reduce sitting time, improve physical activity at work and promote health benefits [25–27], it might also lead to changes in cognitive functions such as productivity [22]. Even though non-significant changes in attention have been found [28], fine motor functions (e.g. mouse moving) as well as mathematical problem solving can be negatively influenced by additional body movements [29]. As studies reporting deterioration of work-related outcomes were all of short duration, studies using long-term follow-up were recommended [22].

The occupational hazards associated with prolonged sitting are receiving renewed attention, and new technologies, devices, and workplace controls to help reduce sitting time are being developed and introduced regularly. It would benefit researchers, practitioners, and employers if these devices and controls were evaluated and studied using consistent and reproducible methods. Reliable and comparable information produced from similarly designed studies would help to separate fact from fiction in the efforts to reduce chronic sitting in the workplace.

### Objectives

The primary objective of this paper is to describe and discuss the methods of a study designed to evaluate the long-term effect of a novel two desk sit-to-stand workplace on sitting time as well as physiological and cognitive parameters for healthy people of working age in comparison to their traditional workplace (control). A secondary objective is to propose methods for future studies of sit-to-stand equipment and intervention programs.

### Hypothesis

The primary hypothesis of the described study is that the ACTIVE OFFICE two-desk sit-to-stand workplace are more effective in reducing occupational sitting time than conventional one desk solutions. Secondary hypotheses are that people using the ACTIVE OFFICE setup will experience positive long term effects on physiological and cognitive skills. The experimental groups received a two desk sit-to-stand workstation in their regular office environments.

## Methods/Design

**Fig. 1:**
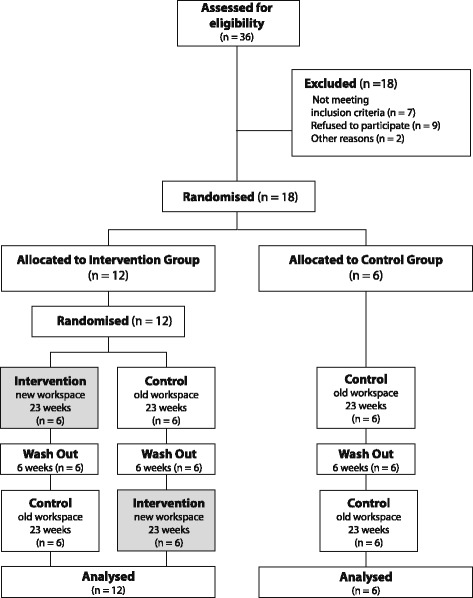
ACTIVE OFFICE study design flow chart

ACTIVE OFFICE is a three-arm randomized control trial with two intervention and one control group (Fig. 1). After the baseline assessment was completed, the participants were randomly allocated to either the intervention or the control arm in a 2:1 ratio. The experimental group subjects received a novel two desk sit-to-stand workstation in their regular office environments, while the control group subjects did not encounter any change in their regular office environments. A 6-week wash out phase was implemented to encourage similar starting conditions for each participant (i.e. using a traditional workplace prior to pre-intervention measurements).

### Participants

A convenience sample of participants was recruited from companies in Linz (Austria) and the surrounding area. A general letter requesting collaboration and providing study descriptions was sent to employers. To reduce recruiting bias, partner-company allocation was randomly done via a regional health insurance provider between August and September 2013. Study details were provided to companies that accepted collaboration in the form of information seminars located at the respective company sites. Separate interviews with people interested in participating in the study took place after the seminars to ascertain the potential subjects’ suitability for study purposes. After exclusion criteria were applied, participants were allocated randomly either to intervention or control groups.

### Subject inclusion criteria

Included subjects were: a) healthy caucasian (no acute or chronic diseases); b) normal weight or slightly overweight (BMI: 18.5–27.5 kg/m^2^); c) aged: 18–60 years; d)regularly working in sedentary office environments; e) regular computer users; f) fluent German speakers; and g) consented to participate.

### Subject exclusion criteria

Excluded subjects had or were: a) heavily overweight & Obesity (BMI >27.5 kg/m^2^); b) short office stay duration (<8 h / day or <20 h / week) c) experience in sit-to-stand workstations; d) acute or chronic diseases; e) inability to stand; f) visual impairments that had not been corrected; g) color blindness h) women who are pregnant or plan to become pregnant within 12 months; i) people planning to change their physical activity level; j) regular smokers (> 1 cigarette /day); or k) not consented to participate.

### Randomization and blinding

After the baseline assessment was completed, the participants were randomly allocated to either the intervention or the control (no intervention) arm in a 2:1 ratio (Fig. 1) by means of a covariate adaptive randomization [30]. Based on previous findings [31], ‘company’ has been determined as a stratum and thus participants were balanced across companies (i.e., 3 participants for each company). On a second level, intervention participants were assigned to either the first (intervention first) or the second (control first) intervention group. Due to the nature of the intervention, participants were not blind to their allocation.

### Sample size

A pilot study with 5 participants, performed in order to estimate the potential of the two-desk setup, found a 22 % reduction of sitting time [32]. Converted to a regular 8-h work day this results in 105 min of sitting time reduction. As this effect was noticeably higher than the effect shown by existing meta-analysis [22] we decided to detect a value between those limits. Therefore, 12 subjects would be needed to detect 90 min differences in sitting time, assuming an alpha risk of 0.05 and beta risk of 0.20 in a two-sided test, and with 20 % loss to follow-up.

### Screening

Study eligibility was determined in private interviews prior to the study. Age, body weight, stature, gender, physical and mental well-being, smoking habits, chronic and acute complaints, pregnancy, medical limitations, medication, working hours per day and week, main occupation and company affiliation were collected via a self-administered questionnaire.

### Intervention for experimental Group

**Fig. 2:**
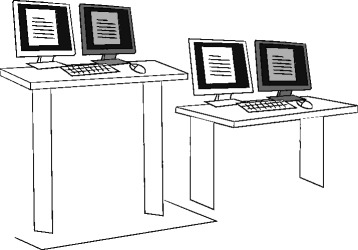
ACTIVE OFFICE two desk sit-to-stand intervention setup

Figure 2 shows the ACTIVE OFFICE two desk sit-to-stand intervention setup. It consists of two equal height-adjustable desks standing next to each other. Precise table arrangements (e.g. 90, 135 and 180°) were self-determined by the participants. To ensure equal conditions, every desk was furnished with the same amount and style of mice, keyboards and screens. Depending on their pre-intervention working conditions, the participants used either one or two screens per desk. The ACTIVE OFFICE was installed 1 day prior to the intervention period at the location of the old desk. Together with the study leader, desk heights were adjusted to the desired sitting and standing heights. Additional software tracking hardware inputs on the standing or sitting desk were installed. The traditional desks were moved to storerooms at the local facility for the duration of the intervention period. During the control phase for the experimental group, both desks were fixed to the sitting height to simulate regular sitting environments. This strategy was used to reduce reconstruction work efforts when the experimental group switched from intervention to control, or vice versa. Reasonable care was been taken to ensure that both desks were equally furnished to avoid preferential effects during both the intervention and control phases (e.g. comparable construction and style of desk, identical equipment and furnishings).

### Control group

Control group subjects did not encounter any changes in their regular office environments.

## Outcome measures

Measurements were made both in the field and in a laboratory. Field measurements were made and processed continuously over the 23-week intervention period. Laboratory measurements were made on two different days, 1 day prior to intervention, and 1 day following intervention (due to cross-over design, each subject underwent 4 total days of laboratory measurements). Field measurements were collected automatically at the participants’ workstation in their working office. Laboratory tests were conducted in a controlled, simulated work-space located at the University of Applied Sciences Campus Linz.

### Outcomes

Primary outcomes were changes in sitting time after 23 weeks in the experimental group compared with its own control period and the control group.

Secondary outcomes within the experimental group were changes in: reaction time, working speed, level of attention, workload perception, physical activity and postural changing pattern for the intervention phase as compared to the control phase.

Tertiary outcomes were changes in salivary cortisol level and heartrate variability (HRV) within the experimental group.

### Experimental group: field measurements

Logging-software was installed on each participant’s computer. By recognizing hardware (mouse, keyboard) inputs on either the sitting or standing desk, the software could determine the proportion of time that a subject was standing versus sitting during the 23-week intervention period.

### Experimental group: laboratory measurements

All laboratory measurements were made in a controlled laboratory at the campus site Linz of the University of Applied Sciences Upper Austria. Temperature, air flow, humidity, lighting conditions (artificial light only) and noise level were controlled and set to be consistent with the subjects’ typical working environment.

Participants were asked to refrain from exercise, caffeine and alcohol and undue stress for 24 h prior to laboratory testing. Food intake 90 min prior to the experiment was prohibited. Subjects were instructed to pursue their usual professional activity in the morning, followed by a laboratory visit in the early evening. To avoid daily fluctuations on performance all measurements started between 1:30 and 2:45 pm.

During the laboratory measurements, subjects either stood or sat upright in an ergonomic office chair, according to the study protocol. Subjects were encouraged to work as fast and as accurately as they could. To ensure identical testing conditions between subjects and to not unduly influence physiological parameters such as salivary cortisol level or heart rate variability, subjects were required to minimize excessive movement (e.g. standing up during the sitting periods). During regular breaks subjects were allowed to visit the toilet. Minor body movements, which typically occur under normal working conditions, were allowed.

### Reaction time, attention & working speed

Physical efforts when performing standardized tests (e.g. standing or walking) can negatively influence cognitive parameters [28, 29]. As studies reporting deterioration of work-related outcomes were all of short duration [19] and there are indications that these are caused by non-familiar working conditions, long-term effects on cognitive performance remain to be identified. Within the study protocol, three different performance-related tests were implemented:

A digital text editing task encouraging participants to fill in spaces in an ergonomic guideline text for 10 min was used. A Stroop-Color-Word-Conflict (Stroop) test, used to measure selective attention and processing speed [33–35] as well as a “d2R-test of attention” (d2R), commonly used in the European area to determine concentration performance [36–38], were implemented.

The simplicity of the text editing task (that did not require any disciplinary knowledge) enabled working speed measurements and simulated typical low effort office work. The implemented digital Stroop-test version contained 190 congruent, incongruent and neutral tasks and required approximately 10 min to simulate long-lasting monotonous office screen work. The d2R-test was executed as a pen and paper version. Therefore, it enabled screen breaks during the test protocol and simulated paper-related office work.

The Stroop-test and the d2R-test are both characterized by a high test-retest reliability (*r* = 0.77–0.95) and do not require any specific previous knowledge except of rudimentary language skills [39, 40]. Normative values for the d2R-test are available for different countries [39].

### Workload perception

Sit-to-stand workstations can evoke positive as well as negative associations [22]. While additional physical efforts caused by standing can lead to higher discomfort especially in the lower extremities (e.g. leg swelling) [22], novel working environments can improve mental well-being [41]. A common method to rate workload perception is the NASA-TLX questionnaire [42]. For reasons of simplicity and unmodified sensitivity [42], the short version of this questionnaire (RTLX), consisting of six major items, was used. Influences on workload perception based on unweighted items in the RTLX were negated due to the cross-over design.

### Salivary cortisol level

Although there are new findings related to the metabolic risks associated with postural changes (breaks in sedentary time) [16, 43, 44], the effect on stress-related parameters is still unclear. Modified cortisol levels after implementing a novel workplace have been shown but the effect of postural changes on cortisol level is not yet known [41]. Therefore, salivary cortisol level was measured during the study protocol and on the following morning in order to detect the cortisol awakening response (CAR) [45].

### Heartrate variability

Heartrate variability (HRV) can be used for predicting all-cause mortality and characterizing cardiovascular health [46, 47]. Improvements of HRV caused by additional physical effort have been shown mainly in physical training programs with medium or vigorous intensity [48–50]. Additional weekly metabolic efforts around 1000 METmin at low intensity level (walking) have also demonstrated positive changes in HRV [51]. Since additional standing (caused by occupational sitting time reduction) should lead to the same level of physical effort (assumption 20–30 % standing), any effect on HRV would be detectable. The 30 min breaks within the study protocol as well as nocturnal periods were used to compare HRV under bias reduced conditions. According to the HRV guideline [46], 24 h Holter monitoring measurements have been implemented. HRV will be analyzed using the software Kubios [52].

### Controlling for outside of work physical activity and sedentary behavior

Physical activity and sedentary behavior are related to physiological and cognitive changes [53, 54]. To avoid bias these parameters have to be determined. The International Physical Activity Questionnaire (IPAQ) has been shown to be reliable and valid for estimating physical activity and sitting time without any further measuring device [55–57]. The long version of this questionnaire (IPAQ-long) additionally enables it to distinguish between occupational and non-occupational activity. To adjust for outside of work sedentary behavior and/or physical activities, the IPAQ-long was interview administered at the beginning of each laboratory measurement day.

### Body movements

Body movements can alter physiological parameters and cognitive performance [28, 58]. Especially small movements during longer time intervals are very hard to classify my means of personal observations. Therefore, a three-dimensional accelerometer – placed on the sternum via a neoprene breast belt – was used to objectively measure body movements. Upper body placements of accelerometers have been shown to reliably detect body movements, and sit-to-stand as well as stand-to-sit transitions [59, 60]. To reduce the total number of sensors, a HRV-recorder with integrated 3D-accelerometer was used (model: medilog AR12 plus, Schiller AG, Baar, Switzerland).

### Measurement protocols

**Fig. 3:**
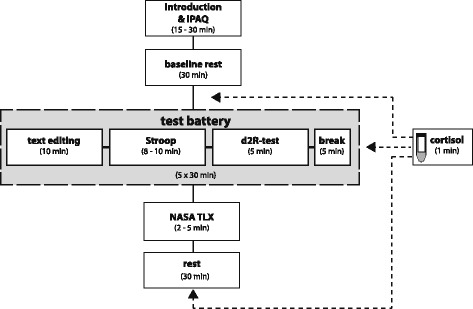
ACTIVE OFFICE study protocol (laboratory measurement)

To test the study hypotheses, several parameters were defined and/or measured under standardized (laboratory measurements) and real life (field measurements) conditions (Table 1). Whereas body postures as well as postural changes were collected continuously during the 23-week intervention period, all further parameters were selectively measured before and after the intervention. To guarantee similar test sequences for each participant, a study protocol for laboratory measurements was developed (Fig. 3), consisting of three phases collecting physiological and cognitive parameters.

**Table 1:** Parameters measured within the ACTIVE OFFICE study

Measurement			Location		Data points	Sampling rate
Parameter	Method	Performed by	Laboratory	Office	d^-1^ (overall)	s^-1^
*Physiological*						
Sitting time	IPAQ-long	questionnaire - interview	x		1 (4)	n.a.^d^
Physical activity	IPAQ-long	questionnaire - interview	x		1 (4)	n.a.^d^
Mental workload	NASA-TLX	questionnaire	x		1 (4)	n.a.^d^
Salivary cortisol	saliva collection	Salivette + cortisol ELISA	x^a^		8 (32)	n.a.^d^
Heart-rate	ECG	ECG recorder	x^b^		n.a.^c^	250
Body movements	acceleration	ECG recorder	x^b^		n.a.^c^	250
*Cognitive*						
Working speed	text editing task	computer software (matlab)	x		5 (20)	>1000
Reaction time	Stroop-test	computer software (matlab)	x		5 (20)	>1000
Attention	d2R-test	test sheet	x		5 (20)	n.a.^d^
*Office based*						
Body postures	logging tool	computer software (C#)		x	n.a.^c^	>1000
Postural changes	logging tool	computer software (C#)		x	n.a.^c^	>1000

^a^Seven measurements in the laboratory followed by one measurement at home on the following morning

^b^Measurement starts in the laboratory and ends at home on the following morning

^c^Data points depending on duration of measurement

^d^Non-digital measuring method (pen & paper)

In the first (initial) phase, participants were familiarized with the study protocol. Sitting time and weekly physical activity were determined via the IPAQ-questionnaire. Examples of each cognitive test implemented in the cognitive phase were executed according to their guidelines [39]. A 30 min break in a sitting posture was used to ascertain baseline heart-rate and cortisol level. Baseline heart-rate was calculated after a 20 min rest for a 5 min interval and saliva samples were collected at the end (30 min) of the break.

In the second (cognitive) phase subjects participated in a test battery containing five blocks. Each block consisted of a working speed test (text editing task), an attentional test (d2R-test of attention) and a reaction time test (Stroop-test). These tests lasted for 30 min to fulfill recommendations regarding postural changes [24]. To simulate “common” working conditions (computer based and non-computer based tasks), digital (text editing task, Stroop-test) as well as pen & paper (d2R-test) versions of the implemented tests were used. All blocks were executed in alternating postures (sit – stand – sit – stand – sit) and at the end of each block – after a 5 min break – salivary samples were collected. The order of posture was not changed within groups or time.

In the third (final) phase participants were asked to estimate their workload by means of the NASA-TLX questionnaire followed by a 30 min resting phase in a sitting posture. During both 30 min resting phases (initial & final) participants watched documentaries and were encouraged not to talk.

Salivary samples were collected after each break during the study protocol and on the following morning, 20 min after waking up, to ascertain cortisol awakening response (CAR). Salivary samples were centrifuged and stored at -80 °C for subsequent testing using a chemiluminescent immunoassay.

Heart-rate was measured from the start of the study protocol until the CAR measurement.

### Statistical analysis

Statistical analyses will be conducted using SPSS version 21 for windows (SPSS, Chicago, IL). Standard statistical methods will be used for calculations for means and standard deviations. Pre- and post- intervention differences will be calculated for sitting time and physical parameters. Paired t-tests will be used to show differences between pre- and post- conditions when the normality condition is satisfied. If not, Mann-Whitney-U tests will be used for pre-post comparison. For cognitive parameters, ANOVA with repeated measures will be used to test whether the different conditions have any effects on the outcome parameters assessed. To reduce learning effects the first block of the test-battery will be ruled out for analysis. When appropriate, post-hoc analyses will be conducted. The effects of time, group and interaction between both variables will be evaluated. To test for normality and homogeneity of variance, Shapiro-Wilk-test and Levene-test will be used, respectively. In general, two-sided tests with an alpha risk of 0.05 and a beta risk of 0.2 are to be accepted.

## Discussion

The ACTIVE OFFICE study evaluates the effects of a novel two desk sit-to-stand workplace on occupational sitting time for healthy office workers. Secondary and tertiary outcomes will deliver insights in physiological and performance-related changes. To our knowledge, a workplace intervention consisting of two equally furnished height-adjustable desks has not been investigated to date.

This study design and approach has several strengths, including the randomized controlled trial design, statistical power analysis, strong inclusion criteria, identical environmental measurement conditions and objective assessment of the primary outcome based on a pilot study.

The study includes recruitment of several different companies to convey a greater pool of people with ergonomic ideas and provide insights into typical Austrian office workplaces. The resulting multisite bias has been reduced by a randomization stratum. The study’s inclusion criteria support a homogeneous collective and will fortify findings. The robust nature of this study design is expected to provide insights into benefits of a two desk sit-to-stand setup. These methods could be employed to study other specific sit-stand interventions or strategies in a robust way.

There are some noteworthy limitations of this study design. For example, as a result of the nature of the intervention it was not possible to blind subjects, although the researcher responsible for the statistical analyses will be blinded. Another limitation is the repeatability of the implemented cognitive tests. Although evaluations regarding short-term reliability have been executed [39, 61], the learning effect resulting from multiple repetition of the “d2R-test of attention” is unknown. Furthermore, as the implemented tests were evaluated in sitting postures only [39], the short-term effect of alternating postures on the performance (e.g. less performance caused by unfamiliar working posture) creates an additional bias. To reduce this bias, a short-term study implementing the ACTIVE OFFICE study protocol has been performed, but data have not yet been analyzed.

There are some additional limitations that are specific to the ACTIVE OFFICE study but these could be easily overcome for future studies. First, due to limitations of hardware input detection, worker idle time (e.g. reading a document or leaving the workstation for a break) could not be directly measured. Sophisticated algorithms can be used to determine whether gaps between hardware inputs should be classified as sitting or standing, but these are imperfect (e.g. 1 min idle time between two sitting periods leads to the conclusion that the subject was sitting the whole time period). Hence, proximity sensors are proposed to be used in future studies to identify associated working postures and idle times more precisely.

Second, the sample size for the ACTIVE OFFICE study is small, and thus statistical power might be limited. Researchers will be able to use the forthcoming results of the ACTIVE OFFICE study to determine appropriate sample sizes for future studies.

This study design is intended to quantify the short to mid-term benefits of using a sit-stand intervention device of strategy. As specified, the design cannot draw conclusions about the long-term sustainability of any measured differences in behavior or performance, nor any long-term health outcomes associated with the changes. Multi-year, prospective studies are needed to test the efficacy of sit-stand technologies, devices, and administrative strategies. Nevertheless, the study design described here provides a repeatable, minimally biased approach to determine what devices and/or strategies have the potential to alter worker behavior and provide positive health benefits.

If the ACTIVE OFFICE setup proves to be successful intervention, it has potential to be implemented in common workplaces. This is crucial since alternating postures as well as reduction in prolonged sitting can promote health benefits and prevent several diseases [16, 20, 43, 44]. Healthy individuals will likely also exhibit less absence time (increase in performance) which in turn leads to decreased health care system costs. If cognitive performance improvements can be shown, additional costs for a two desk setup will become more acceptable.

The methods used for the ACTIVE OFFICE study are generalizable and can serve as a common foundation upon which future studies can determine the potential efficacy of sit-stand devices and strategies. If future studies employ substantially similar methods, the results between studies would likely be directly comparable and this would help employers, practitioners, and future researchers to design appropriate sit-stand interventions.

## Trial status

The recruitment for the “ACTIVE OFFICE” trials was initiated during August – September 2013. The baseline measurements and the post intervention measurements (23 weeks after baseline) were completed in September 2014 and April 2015, respectively. The study is currently at the stage of data analysis.
